# Assessment of the mechanical performance of a novel CT-linac treatment couch

**DOI:** 10.3389/fonc.2026.1773010

**Published:** 2026-05-01

**Authors:** Linyi Shen, Mengyang Li, Guiyuan Li, Hualin Wang, Jialin Ding, Yunxiang Wang, Xuejie Xie, Siqi Yuan, Yujie Kang, Junfeng Qi, Xinyuan Chen, Shouping Xu, Jianrong Dai, Yuan Tian

**Affiliations:** National Cancer Center/National Clinical Research Center for Cancer/Cancer Hospital, Chinese Academy of Medical Sciences and Peking Union Medical College, Beijing, China

**Keywords:** ct-linac, IGRT, mechanical performance, quality assurance, treatment couch

## Abstract

**Purpose:**

This study aims to systematically evaluate the mechanical performance of the novel treatment couch for the uLinac VisionaryTx CT-Linac (United Imaging Healthcare, China).

**Materials and methods:**

Quality assurance protocols were developed by integrating AAPM TG-142 and TG-66 guidelines. The following key parameters were assessed: (1) couch top movement accuracy, including translational/rotational precision and perpendicularity to the CT imaging plane; (2) couch top rigidity under varying loads (0–135 kg) and extensions; (3) couch base movement accuracy, covering longitudinal travel reproducibility and rotational homing; (4) efficacy of the laser rangefinder-based sag correction and the stability of the rangefinder itself; and (5) comprehensive end-to-end evaluation of IGRT workflows to determine the overall impact of couch mechanical performance on delivery accuracy. All tests were conducted under clinically representative loaded conditions.

**Results:**

The couch top demonstrated high accuracy in translational movement (≤ 0.5 mm), rotation (< 0.12°), and perpendicularity relative to the CT imaging plane. Under maximum load, the couch top showed a sag of 4.1 mm, demonstrating superior rigidity to a dedicated CT simulator while being slightly less rigid than conventional linac couches. The couch base achieved precise longitudinal movement (≤ 1 mm) and rotational homing accuracy (< 0.1°). The laser rangefinder-based correction method achieved good agreement between calculated and measured height variations (deviation < 0.6 mm) with excellent stability in both angular and distance measurements. End-to-end tests verified sub-millimeter (0.5 mm) and sub-degree (0.5°) accuracy for IGRT workflows across various loading conditions.

**Conclusion:**

The novel CT linac couch system (uLinac VisionaryTx) exhibits exceptional mechanical performance and fully satisfies the precision requirements for CT acquisition, image guidance, and setup error correction.

## 1 Introduction

The CT-Linac is a novel radiotherapy system characterized by its integration of a diagnostic-quality helical computed tomography (CT) scanner directly behind the gantry of a C-arm linear accelerator (linac). This integrated architecture supports a seamless workflow from simulation to treatment within a single unit, reducing both the system’s physical footprint and idle time, thereby offering a cost-efficient solution for small to medium-sized medical facilities (1). Moreover, the integrated kilovoltage fan-beam CT (KV FBCT) is less susceptible to photon scattering compared to the kilovoltage cone-beam CT (KV CBCT) commonly employed in image-guided radiotherapy (IGRT) (2). As a result, it yields high-quality CT images with fewer artifacts and enhanced uniformity (3). This technical superiority confers two key clinical advantages: First, when CT images acquired prior to a treatment fraction are co-registered with the planning CT, superior image quality leads to improved registration accuracy and, consequently, more precise correction of patient setup errors (4); Second, the accurate CT number provided can be directly utilized for delineating target and organ at risk, optimizing treatment plans and performing dose calculations, thereby facilitating the online adaptive radiotherapy and the implementation of so-called all-in-one radiotherapy, particularly for emergency radiotherapy (5).

In conventional medical linac, the treatment couch is typically designed to support and immobilize the patient during treatment and perform only minor translations (~cm) and/or rotations (~3 degree) for setup errors correction. In contrast, the treatment couch system in a CT linac not only fulfills these roles but is also capable of moving the patient over a distance exceeding 2000 mm between the radiotherapy treatment position (RT center) and the CT imaging position (CT center) through coordinated motion of the couch base and couch top. Additionally, at the CT center, the couch top enables continuous longitudinal motion to facilitate the acquisition of helical CT images.

Given these expanded functionalities, the mechanical performance of CT linac’s treatment couch exerts a more significant influence on treatment accuracy and demands rigorous quality assurance (QA). To date, few studies have addressed the commissioning and QA for earlier version of CT linac, such as uLinac 506c. For instance, Yu et al. and Wen et al. assessed the rotational isocentric alignment among the gantry, collimator, and treatment couch systems (6, 7), while Sun et al. evaluated the translational and rotational accuracy for the treatment couch under no-load conditions (8). However, these conventional QA protocols, largely derived from the AAPM TG-142 report (9), are inadequate for ensuring the accuracy of CT linac. Notably, they lack specific QA test relevant to KV FBCT imaging, such as verifying the perpendicularity between the longitudinal motion of treatment couch and the CT imaging plane.

In 2024, a new generation of CT linac (uLinac VisionaryTx, United Imaging Healthcare, Shanghai, China) was installed in our hospital and prepared for clinical operation. It incorporates several key enhancements to its treatment couch. The longitudinal travel range of the couch base has been extended to 2106 mm, enabling patient shifting between the RT and CT centers using solely the couch base longitudinal movement, without requiring coordinated couch top longitudinal movement. Moreover, a 6-degree-of-freedom (6D) couch top has been incorporated to improve the correction of patient positioning errors. These new capabilities impose updated requirements for the QA of the couch system’s mechanical performance, such as comprehensive evaluation of the 6D couch top’s translational and rotational accuracy.

This study presents the first systematic assessment of the mechanical performance of the uLinac VisionaryTx (United Imaging Healthcare, Shanghai, China)’s treatment couch. To clarify the rationale behind each QA item, this paper begins with a concise overview of the uLinac VisionaryTx system’s basic structure and clinical workflow. An analysis of the error sources affecting the treatment accuracy related to the couch mechanical performance was conducted to identify key parameters requiring QA. To distinguish our work from existing literature, we subsequently focus on QA items that are either underreported in published studies or specifically tailored to the new system’s advanced functionalities. Corresponding results for each QA item are then presented to serve as a reference for QA of similar linac systems.

## 2 Material and methods

### 2.1 Brief introduction of CT linac and its clinical workflow

**Figure 1:**
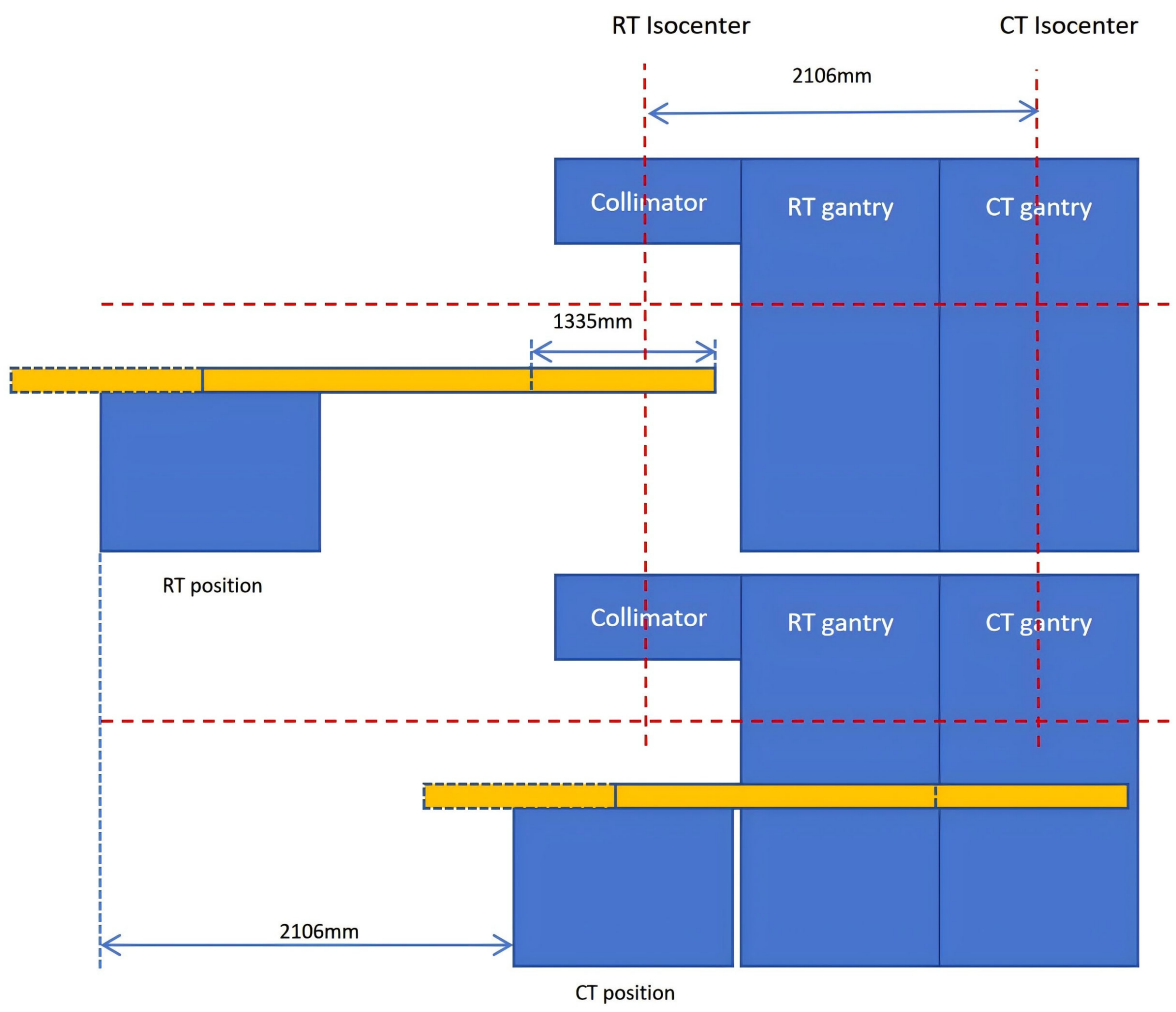
Structural diagram of CT-Linac.

The uLinac VisionaryTx linac, features an innovative coaxial integration a 40-slice spiral CT (bore size: 87 cm) with a C-arm linac, as illustrated in **Figure 1**. The CT system is positioned 2106 mm away from the RT center. The treatment couch system consists of a movable base and a couch top with a load capacity of 190 kg. The couch base provides 2106 mm of radical movement and isocentric rotation capability. The couch top allows three-dimensional translations, including 1335 mm of longitudinal movement, and rotational adjustments in pitch and roll axes (± 3 degrees) for patient setup correction. The angle sensors of the couch top are installed at the rear of the couch top for positional feedback.

**Figure 2:**
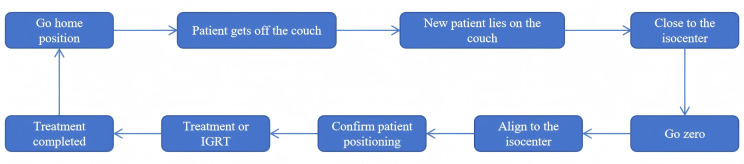
The flow chart of the clinical workflow.

**Figure 2** outlines the clinical workflow, with the detailed procedures as follows. First, the therapist activates the “Go Home Position” function to return the couch’s isocentric rotation angle to zero and moves the lateral, longitudinal, and vertical axes to a preset location that facilitates patient access. After assisting the new patient onto the couch and approximating the isocenter, the “Go Zero” function resets the pitch and roll angles back to their zero positions. Once the patient’s surface markers are precisely aligned with the room lasers via fine manual couch adjustments, the “Setup Confirm” button is clicked to finalize the setup, and treatment or the IGRT procedure can be initiated according to the clinical protocols. After treatment, the “Go Home Position” function is used again to return the couch to the safe position, facilitating patient disembarkation.

Upon confirmation of setup, the acquisition procedure of the integrated FBCT can be initiated. Then the couch will automatically move to the predefined preparation position (Z = -15 cm, X = 0 cm, with other axes maintaining their positions) to prevent potential collisions with the gantry during subsequent longitudinal movement of both the couch base and top. Finally, the patient is transported from the RT center to the CT center via longitudinal movement of the couch base, followed by continuous longitudinal motion of the couch top to complete the CT acquisition.

### 2.2 Analysis of error sources

First, the uLinac VisionaryTx system acquires CT images through longitudinal movement of the couch top at the CT center. Therefore, in accordance with AAPM TG-66 recommendations (10), the perpendicularity between the couch top longitudinal motion and the imaging plane should be verified to prevent CT image distortion. Additionally, as positioning error corrections are achieved through rotational and translational movements of the couch top, the accuracy of all these movements directly influences the overall treatment precision.

Second, in clinical practice, planning CT acquisition may be performed using either the system’s integrated CT or dedicated CT simulators. This operational flexibility necessitates comparable couch top rigidity across the uLinac VisionaryTx, dedicated CT simulators, and conventional linacs. Otherwise, treatment fractions delivered without image guidance may exhibit systematic pitch error between simulation and actual treatment delivery, even following initial isocenter alignment using external lasers and skin markers. These angular deviations would introduce clinically significant displacement errors at both the superior and inferior extremities of an elongated target volume. For instance, with the treatment isocenter located at the target center, a mere 0.3 degree pitch error can result in a displacements exceeding 1 mm at each end of a 40 cm-long target (11, 12). Therefore, both couch rigidity and the accuracy of the digital indicator represent critical QA items, as recommended in the AAPM TG-142 report.

Third, the uLinac VisionaryTx switches patients between the RT and CT centers through a large-range longitudinal movement (~2106 mm) of its couch base. This necessitates stringent verification of geometric accuracy for couch base in two aspects: the perpendicularity of the couch base’ longitudinal travel relative to the CT imaging plane when the couch base isocentric rotation is at 0 degree, as well as the repeatability of its rotation homing precision. Deviations in these parameters may induce spatial distortions in reconstructed CT images. Additionally, accuracy and repeatability in the couch base longitudinal movement may introduce systematic registration errors that propagate through the treatment workflow.

**Figure 3:**
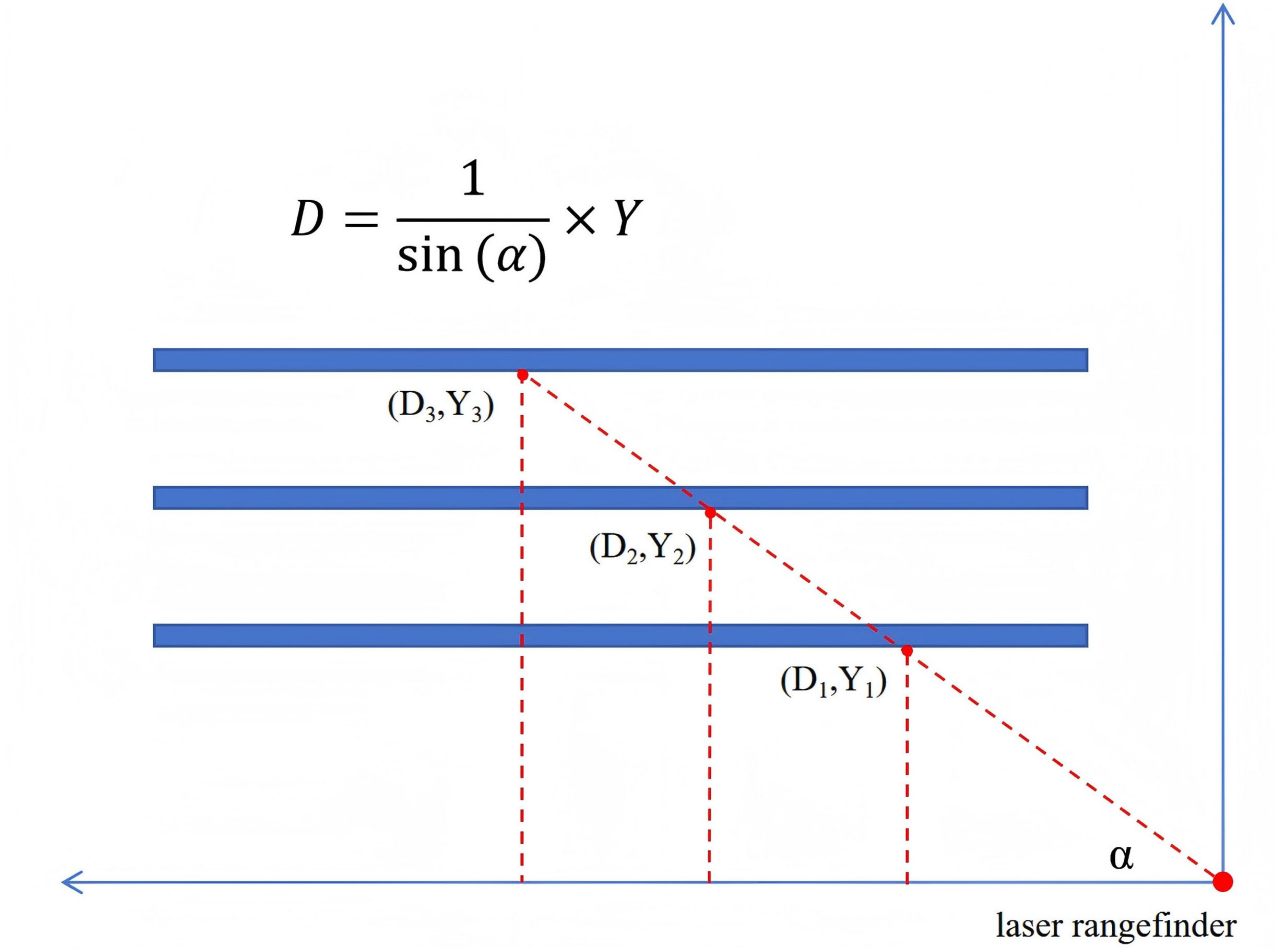
Schematic for angular calibration of laser rangefinder.

**Figure 4:**
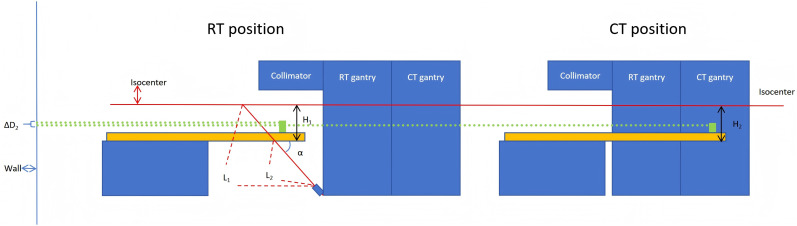
Schematic diagram of sag correction for the Treatment couch between CT and RT center.

Fourth, the inevitable sag of couch top between RT center and CT center is another critical source of registration error. The magnitude of sag is modulated by multiple mechanical factors, including applied load, extension length, and the levelness of couch base longitudinal movement. To address this challenge, United Image Healthcare company (UIH) has implemented an automated sag compensation strategy incorporating a floor-mounted laser rangefinder beneath the gantry. The device projects a laser beam at a fixed angle α to monitor the distance to the couch top’s lower surface. During system calibration, the couch is positioned at three different height levels (shown in **Figure 3**), with the acquired distance measurement enabling precise determination of sin(α) through least-squares fitting. Additionally, by positioning the upper surface of the couch at source-to-surface distance (SSD) = 95 cm, and considering the couch top’s 5 cm thickness, its lower surface aligns precisely with the isocenter plane (SSD = 100 cm, as shown in **Figure 4**). The distance L_1_ measured by the laser rangefinder to the lower couch surface at this position is recorded as a system parameter and utilized for sag compensation during clinical treatment delivery.

During the clinical workflow, after confirmation of patient positioning and initiation of CT image acquisition, the system automatically records the current couch coordinates and transitions the treatment couch to the preparation position. The laser rangefinder subsequently measures distance L_2_ to the lower surface of the couch top at this location. The vertical distance H_1_ from the couch top’s lower surface to isocenter under current conditions is calculated as:

(1) $$H_{1}=(L_{1}-L_{2})\times \sin(\alpha )$$

While maintaining this couch height, the patient is moved to the CT center via couch base’s longitudinal movement for CT acquisition. The system automatically identifies the couch top’s lower surface in the reconstructed CT images and calculates the vertical distance H_2_ from the CT scanning center to the couch top’s lower surface. The discrepancy between H_1_ and H_2_ (denoted as ΔD_1_) represents the systematic vertical deviation introduced by couch sag between CT and RT centers. This systematic vertical deviation is subsequently applied as a compensatory correction during image registration. Therefore, the accuracy of the laser rangefinder and the stability of angle α are critical for reliable correction of this systematic sag deviation.

Finally, comprehensive end-to-end tests were performed across all available IGRT workflows (KV FBCT and MV CBCT) on the uLinac VisionaryTx system to evaluate the overall impact of treatment couch mechanical performance on delivery accuracy.

It should be noted that, since loaded conditions better represent the actual clinical scenario, all subsequent tests were performed under loaded conditions unless otherwise specified.

### 2.3 Accuracy of the couch top movement

#### 2.3.1 Perpendicularity of the couch top’s longitudinal movement with respect to the CT imaging plane

The perpendicularity of the couch top’s longitudinal movement with respect to the CT imaging plane was evaluated following the methodology provided in Appendix E part (1) of the AAPM TG-66 report. The difference in the location of the center hole in the laser QA device, imaged at the head and foot of the couch top, should be within 2 mm.

#### 2.3.2 Directional and distance accuracy of the couch top movement

The accuracy of the couch top’s translational (lateral, longitudinal, and vertical) movements was evaluated in accordance with Part (2), Appendix E of the AAPM TG-66 report.

Beyond these standard QA tests, particular emphasis was placed on assessing the directional fidelity of each movement axis. The crosshairs of the 40cm × 40cm light field, with the gantry and collimator rotation at absolute zero degrees, were used to establish the X (lateral) and Y (longitudinal) coordinate axes of the accelerator. Graph paper was affixed flat on the couch top’s upper surface and aligned with these crosshairs while the couch isocentric rotation remained at absolute zero. A reference point was marked on the longitudinal (or lateral) axis of the crosshairs at the edge of the light field. The deviation of this point from the lateral (or longitudinal) crosshairs axis was monitored during full-range travel of the couch top in the Y (or X) direction. To assess unintended vertical displacement (Z-direction) during horizontal couch movement, a laser level was positioned on the couch top at the isocenter. The height of its projected horizontal laser line on the wall was compared before and after couch top movement in the Y (or X) direction. Any change in the projected height indicates a Z-axis deviation during the couch top’s horizontal motion.

The rotation (pitch and roll) accuracy of the treatment couch was quantified using a digital inclinometer placed at the isocenter on the couch top. The couch top was incremented from 0° to their maximum range (± 3°) in 0.5° intervals for both rotational axes. At each angle setting, the inclinometer was positioned along the X-axis for pitch measurements and the Y-axis for roll measurements. The readings from inclinometer were then compared against the corresponding digital readouts from the digital indicator to determine measurement agreement.

### 2.4 Rigidity of the couch top

To evaluate treatment couch rigidity, all mechanical components (gantry, collimator, and couch isocentric rotation) were first initialized to their zero positions. The couch height was adjusted to achieve a SSD of 100 cm, with lateral position zeroed. A steel ruler was suspended vertically adjacent to the couch side. Under no-load conditions, the couch top was positioned longitudinally to different locations representing various treatment sites: 40 cm (head), 62 cm (chest), 99 cm (abdomen), and 133 cm (pelvis). At each position, two measurements were taken near the isocenter: couch top inclination using a digital level, and the projection position of the horizontal laser line from a laser level recorded on the vertically suspended ruler. Following no-load baseline measurements, distributed loads of 30 kg, 80 kg and 135 kg were sequentially applied over a 2-meter range on the couch top’s upper surface. Under each loading condition, the identical measurement protocol was repeated to quantify couch top inclination and vertical displacement near the isocenter across all specified positions, serving as metrics for assessing couch top’s rigidity.

Utilizing the methodology described above, we systematically measured and compared the rigidity of the couch top across three systems: uLinac VisionaryTx, dedicated CT simulator (Bigbore RT, Philips Healthcare, Amsterdam, the Netherlands) and conventional linac (Axesse, Elekta, Sweden, Stockholm).

### 2.5 Accuracy of couch base movement

#### 2.5.1 Perpendicularity of the longitudinal movement with respect to the CT imaging plane

Since the couch base cannot perform continuous longitudinal movement for CT image acquisition like the couch top, the method recommended in AAPM TG-66 is not applicable for assessing the perpendicularity of couch base’s longitudinal movement relative to the CT imaging plane at a 0°couch isocentric rotation. As an alternative approach, we assessed the coaxiality between the longitudinal movements of the couch base and the couch top using a method similar to the evaluation of the coincidence of the couch top’s longitudinal motion with the longitudinal axis of the light field crosshairs at 0°couch isocentric rotation. This indirect evaluation is justified by the previously established high perpendicularity of the couch top’s longitudinal movement relative to the CT imaging plane, which serves as a reliable reference.

#### 2.5.2 Repeatability of the couch base isocentric rotation homing

Under the aforementioned test conditions, the treatment couch was rotated to an arbitrary angle and subsequently returned to the zero position via the system’s automatic homing function. Following each isocentric rotation homing procedure, the lateral deviation of the reference point movement relative to the longitudinal axis of the crosshairs, induced by longitudinal motion of the couch base, was recorded using methodology consistent with the approach described in the preceding section.

#### 2.5.3 Accuracy and reproducibility of couch base longitudinal movement

A 140 kg load was uniformly distributed over a 2-meter section of the couch top, with the mass center positioned at the isocenter. The couch height was adjusted to a SSD of 100 cm. A laser level was placed at the isocenter, and the projection points of its crosshairs were marked on the lateral walls and foot-end wall. The couch base was then moved from the RT center to the CT center. The travel distance was measured using a measuring tape. The couch base was subsequently returned to the RT position, and the maximum deviation from the initial marks was documented to assess the positional reproducibility.

### 2.6 Accuracy of sag correction and stability of the laser rangefinder

To validate the accuracy of the UIH-proposed laser rangefinder-based method for correcting couch top sag between RT and CT centers, a steel ruler was vertically mounted on the treatment room wall. The couch was positioned at the preparation position (-15 cm height), and a laser level was placed at the isocenter in the RT position. The position of its horizontal laser line projected onto the ruler was recorded as M1. following longitudinal movement of the couch base to the CT center, the new projection position of the horizontal laser line was recorded as M2. The difference between M1 and M2 (ΔD2) represents the actual vertical displacement of the couch top between the RT and CT centers. Concurrently, the corresponding values of L1, L2, and sin(α) were retrieved in service mode. These values were applied to Equation 1 to calculate the height difference ΔD1, which was then compared with the directly measured ΔD2.

As derived from Equation 1, the stability of the laser rangefinder and the constancy of inclination angle α critically determine the correction accuracy. Therefore, the distance from the laser rangefinder to the couch top’s lower surface at the -15 cm height was measured ten times daily over one month. Monitoring of variation in sin(α) was conducted throughout the same period using the standard calibration protocol.

### 2.7 End-to-end test

Comprehensive end-to-end testing was conducted using an IGRT QA phantom. Planning CTs were acquired utilizing both the integrated CT and a dedicated CT simulator (Big bore RT, Philips Healthcare). To evaluate system performance across clinically representative conditions, scanning was performed under different loading (30 kg for pediatric, 80 kg for standard adult, 140 kg for obese patient) and phantom locations (40 cm for head, 62 cm for thoracoabdominal, and 133 cm for pelvic). For each configuration, the phantom was precisely aligned at the isocenter and scanned using appropriate clinical protocols for head, thoracoabdominal, and pelvic imaging, respectively.

On the linac, the identical loading conditions and phantom locations were replicated. To assess image registration and correction capabilities, the phantom deliberately displaced by 15 mm along the X, Y, and Z direction to simulate setup error. Both MV CBCT and FBCT IGRT workflows were executed following manufacturer-recommended clinical protocols. The system’s automated registration algorithm calculated the positional discrepancy between the planning CT and IGRT images, with expected values approximating the introduced 15 mm setup error. After performing correction based on image registration results by both 3D and 6D movement of couch, the correction accuracy was further verified through visual assessment of the coincidence between the phantom’s central marker and room lasers. To quantitatively evaluate correction accuracy, subsequent MV CBCT scans were acquired to measure residual setup errors.

## 3 Results

### 3.1 Accuracy of the couch top movement

#### 3.1.1 Perpendicularity of the couch top with respect to the CT imaging plane

CT imaging of the Laser QA phantom demonstrated clear crosshairs visualization, confirming precise perpendicularity between the couch top’s longitudinal movement and the CT imaging plane. Under no-load conditions, the measured deviations of the phantom’s center hole were 0.2 mm in the horizontally and 1.1 mm vertically. Under a 135 kg load, these deviations increased marginally to 0.3 mm and 1.3 mm, respectively.

#### 3.1.2 Accuracy in direction and distance of the couch top movement

**Table 1** summarizes the directional and positional accuracy of the couch top’s 3D movements under a 135 kg load. During longitudinal (Y-axis) travel over a 40cm range, the couch top demonstrated perfect positional reproducibility with no measurable deviation. The maximum directional errors were 0.5 mm in the transverse (X-axis) dimension with no vertical (Z-axis) component. For lateral movements within the ± 25 cm travel range, the system exhibited a maximum positional deviation of 0.5 mm, which manifested as a vertical directional error of less than 0.5 mm while maintaining perfect lateral directional fidelity. Throughout the whole vertical travel range (- 63 cm to + 17 cm), the maximum observed positional deviation was 0.5 mm, with corresponding directional errors not exceeding 0.5 mm in both lateral and longitudinal dimensions.

**Table 1:** Accuracy in direction and distance of the couch top translational movement.

Movement direction	Positional deviation (mm)	Directional deviation(mm)		
		X	Y	Z
Longitudinal	0.0	0.0	/	<0.5
Lateral	0.5	/	0.0	<0.5
Vertical	0.5	<0.5	<0.5	/

**Figure 5:**
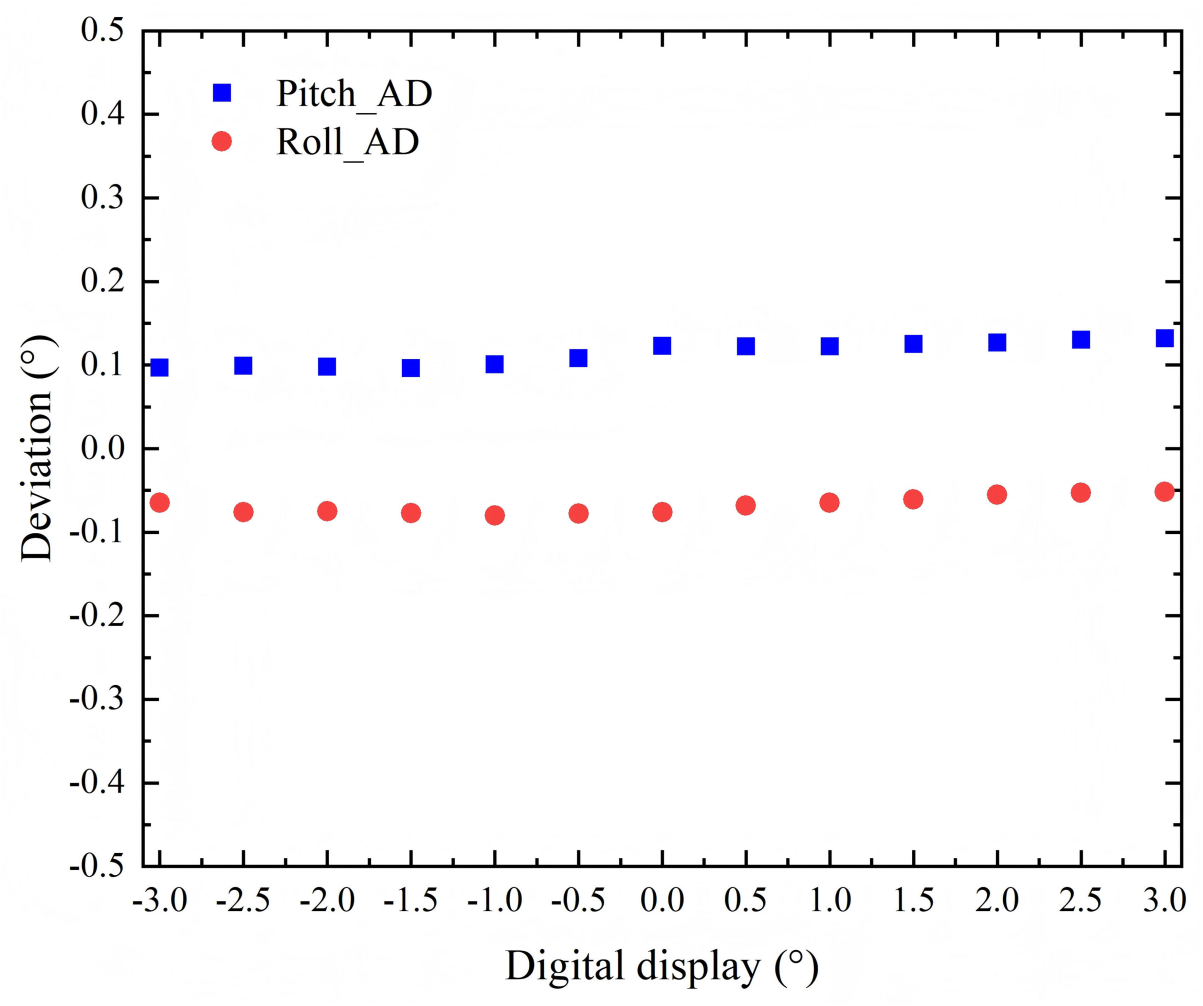
Accuracy of the digital indicator for rotation angle of couch top.

**Figure 5** presents the discrepancies between digital inclinometer measurements at the isocenter and the couch top’s digital indicator reading for pitch and roll angles, obtained under a 135 kg with 40 cm couch extension. Across the full ±3° rotational range, the mean absolute deviations (± SD) were 0.11° ± 0.01°for pitch and - 0.07° ± 0.01°for roll rotations.

**Figure 6:**
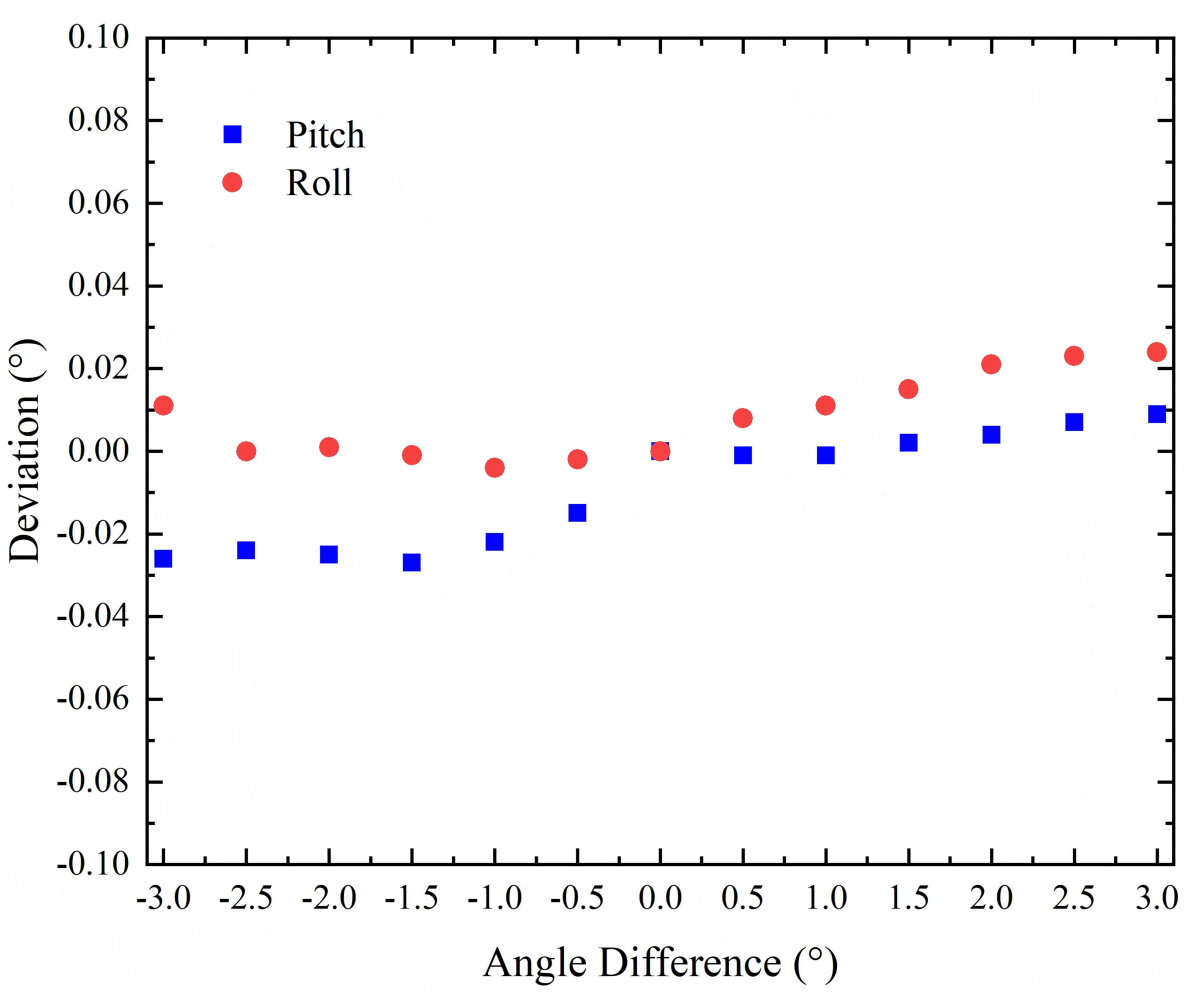
Accuracy of the couch top pitch and roll rotation.

As shown in **Figure 6**, under 135 kg load the discrepancy between the angular variation measured by digital inclinometer at the isocenter and the corresponding change displayed on the couch top’s digital angle indicator remained within 0.03°.

### 3.2 Rigidity of the couch top

**Figure 7:**
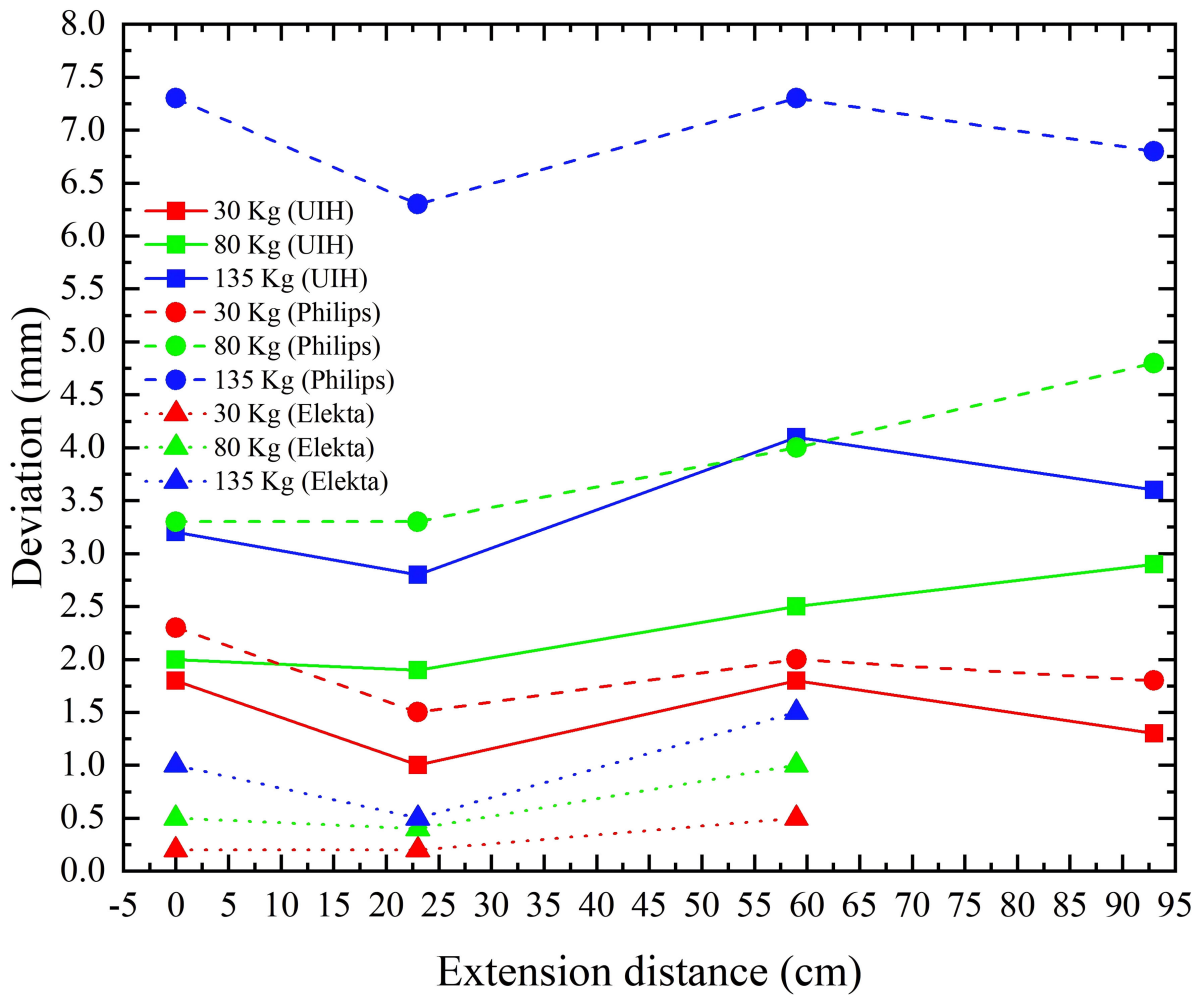
Rigidity of the couch top from different company.

**Figure 7** presents the measured isocenter sag under varying loading conditions and couch extension distances for three systems: a CT linac (uLinac VisionaryTx, UIH), a dedicated CT simulator (Big Bore RT, Philips Healthcare), and a conventional linear accelerator (Axesse, Elekta).

Relative to the baseline unloaded condition, the maximum couch-top sag was 4.1 mm for the CT linac, 7.3 mm for the dedicated CT simulator, and 1.5 mm for the conventional linac. The corresponding maximum change in pitch angle at isocenter remained within 0.25°, 0.3°, and 0.2°, respectively, across all tested conditions.

In all systems, isocenter sag increased with applied load at a fixed extension distance. Under constant loading, the sag varied with extension distance, with maximum variations of approximately 1.3 mm (CT linac), 1.5 mm (CT simulator), and 1.0 mm (conventional linac).

### 3.3 Accuracy of the couch base movement

#### 3.3.1 Perpendicularity of the longitudinal movement with respect to the CT imaging plane

At a 0° isocentric couch rotation, longitudinal movement of the couch top through 40 cm resulted in a maximum lateral deviation of the reference point trajectory from the crosshairs longitudinal axis of less than 0.5 mm. Conversely, longitudinal movement of the couch base across the same distance produced a maximum lateral deviation of less than 0.8 mm from the reference axis. Based on these measurements, the angular divergence between the longitudinal movement directions of the couch base and couch top was calculated using the arctangent function to be less than 0.05°.

#### 3.3.2 Repeatability of the couch base rotation angle retraction homing

Measurement results demonstrate that following automated couch base homing, the lateral deviation between the reference point trajectory and the crosshairs longitudinal axis remained below 0.5 mm, regardless of the initial isocentric rotation angle. This corresponds to a rotational homing accuracy of better than 0.1°for the couch base system.

#### 3.3.3 Accuracy and reproducibility of couch base longitudinal movement

The measured travel distance of the couch base from the RT to CT center was 2107 mm, deviating 1 mm from the preset value. Throughout this travel range, the couch base’s longitudinal movement demonstrated high geometric fidelity, with lateral deviations remaining below 0.5 mm and vertical displacements not exceeding 1 mm. Furthermore, the couch base exhibited exceptional positional reproducibility between the RT and CT centers, showing maximum positioning variations of 0.1 mm.

### 3.4 Accuracy and stability of the laser rangefinder

As presented in **Table 2**, a sag of approximately 1.2 mm was observed between the CT and RT centers even under no-load conditions. This systematic deviation exhibited progressive amplification with increasing load, reaching a maximum of 3.5 mm under 135 kg loading. These findings underscore the critical necessity for implementing corrections during FBCT image-guided procedures.

**Table 2:** Accuracy of laser rangefinder based sag correction method proposed by UIH.

Weight (kg)	H_1_ (mm)	H_2_ (mm)	ΔD_1_ (mm)	ΔD_2_ (mm)	ΔD_1-2_ (mm)
0	200.2	201.7	1.5	1.2	0.3
30	202.1	204.1	2.0	1.5	0.5
80	203.6	206.7	3.1	2.5	0.6
135	204.2	208.2	4.0	3.5	0.5

Validation experiments confirmed that across all tested loading conditions, the laser rangefinder-based correction method developed by UIH achieved good agreement between calculated and measured couch height variations, with maximum residual error less than 0.6 mm.

**Figure 8:**
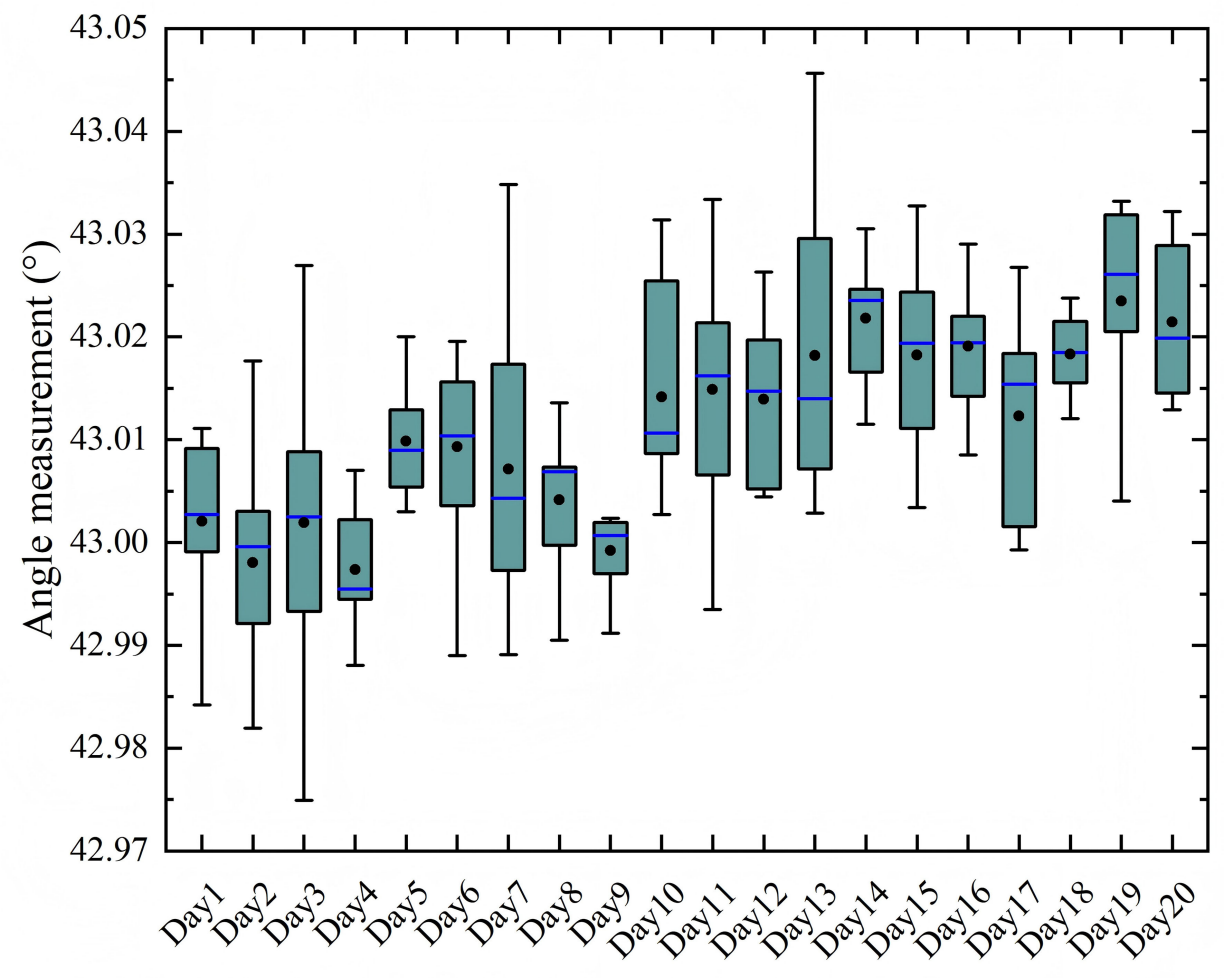
Stability of the laser rangefinder’s angle.

**Figure 9:**
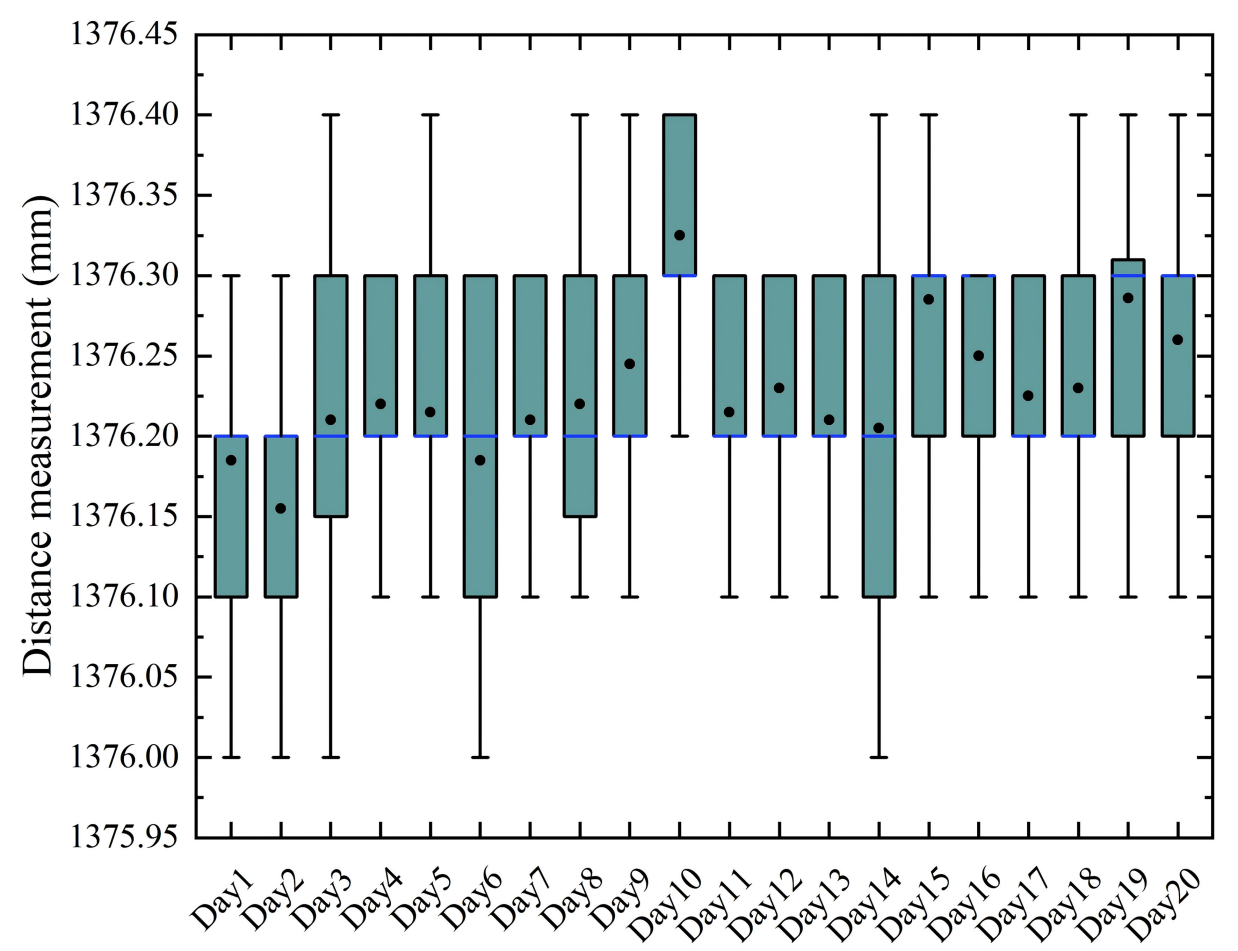
Stability of the laser rangefinder’s distance measurements.

**Figures 8**, **9** present the stability assessment results for the laser rangefinder’s angular and distance measurements, respectively. Throughout the testing regimen comprising 200 measurements (10 daily measurements over 20 days), the measured angle α ranged from 42.97° to 43.04°, with an overall mean and standard deviation of 43.01° ± 0.01°. The maximum daily coefficient of variation for angular measurements was 3.34 × 10^-4^. Similarly, distance measurements demonstrated comparable stability, with values distributed between 1376.00 mm and 1376.40 mm. The overall distance mean and standard deviation were 1376.22 ± 0.08 mm, while the maximum daily coefficient of variation remained at 7.26 × 10^-5^.

### 3.5 End-to-end test

As shown in **Table 3**, comprehensive evaluation of MV CBCT and FBCT IGRT workflows across varying reference CT sources (integrated CT simulator versus dedicated CT simulator) and loading conditions demonstrated satisfied accuracy for intentionally introduced setup errors. Under all clinically relevant scenarios, both MV CBCT and FBCT IGRT workflows successfully identified positional deviations with translational errors not exceeding 0.5 mm and rotational errors within 0.5°. Following 3D/6D correction implementation, subsequent MV CBCT verification and visual assessment confirmed that residual displacements below 0.5 mm and rotational residuals under 0.5°. Notably, comparative analysis revealed superior overall accuracy in IGRT workflows when utilizing UIH’s integrated CT scanner for reference imaging, compared to dedicated CT simulator from other manufacturers.

**Table 3:** Evaluation of MVCBCT and FBCT IGRT workflows using reference CTs from different CT simulator and patient weights.

(a)													
CT simulator_Philips													
Loading (kg)	Position	CBCT						FBCT					
		VRT (mm)	LNG (mm)	LAT (mm)	Yaw (°)	Pitch (°)	Roll (°)	VRT (mm)	LNG (mm)	LAT (mm)	Yaw (°)	Pitch (°)	Roll (°)
30	Couch_head	-14.8	14.9	15.4	0	0.1	-0.1	-15.3	14.9	15.4	0.0	0	-0.1
	Couch_middle	-14.9	15.1	15.1	-0.1	0.2	-0.3	-15.2	14.9	15.1	0.0	0.2	-0.2
	Couch_end	-15.2	14.8	15.1	0.0	0.3	-0.2	-14.9	14.8	14.9	-0.1	0.2	-0.2
80	Couch_head	-15	14.8	15.3	0.0	0.0	-0.3	-15	14.7	14.9	0.0	0.2	-0.4
	Couch_middle	-15.4	14.8	15.1	-0.1	0.4	-0.2	-15	14.7	15.3	-0.1	0.2	-0.2
	Couch_end	-15.4	14.6	15.2	0.2	0.3	-0.4	-15.2	14.9	15.0	0.0	0.2	-0.2
135	Couch_head	-14.9	15.1	15.2	0.2	0.3	-0.4	-14.9	14.8	15.1	0.2	0.2	-0.4
	Couch_middle	-14.8	14.7	15.5	-0.2	0.3	-0.4	-14.8	15.3	15.4	-0.1	0.1	-0.4
	Couch_end	-15.1	14.9	15.2	0.0	0.5	-0.2	-15.0	15.3	15.3	0.0	0.2	-0.1

(b)													
CT simulator_UIH													
Loading (kg)	Position	CBCT						FBCT					
		VRT (mm)	LNG (mm)	LAT (mm)	Yaw (°)	Pitch (°)	Roll (°)	VRT (mm)	LNG (mm)	LAT (mm)	Yaw (°)	Pitch (°)	Roll (°)
30	Couch_head	-15.3	14.9	15.2	0.0	0.0	0.0	-15.1	14.7	15.1	0.1	0.0	0.0
	Couch_middle	-14.8	15.0	15.2	0.0	0.2	0.0	-14.7	14.9	14.7	0.1	0.0	0.1
	Couch_end	-15.2	15.0	14.9	0.1	0.1	0.2	-15.1	14.8	14.7	0.1	0.0	0.2
80	Couch_head	-15.1	14.9	15.1	0.0	0.0	0.0	-15.1	14.8	15.1	0.1	0.0	0.1
	Couch_middle	-14.9	14.8	15.3	0.0	0.2	0.0	-14.6	14.9	14.9	0.0	0.0	0.0
	Couch_end	-15.3	15.1	15.3	0.0	0.1	0.0	-15.1	15.4	15.3	0.0	0.0	0.0
135	Couch_head	-15.2	14.9	15.2	0.0	0.0	0.0	-15.2	14.8	15.2	0.0	0.0	0.0
	Couch_middle	-15.0	15.0	15.0	0.0	0.2	0.0	-15.1	15.2	15.0	0.0	0.0	0.0
	Couch_end	-15.3	14.9	15.2	0.0	0.2	0.0	-15.0	15.2	15.0	0.0	0.0	0.0

## 4 Discussion

The innovative coaxial non-coplanar integration of FBCT with the linac in CT linac, provides superior pre-treatment image quality compared to conventional CBCT. This directly enhances image registration precision and establishes the foundation for clinical implementation of online adaptive radiotherapy. However, it also imposes expanded functional requirements on the treatment couch system. Beyond performing standard function of patient support, immobilization and setup error correction, the couch must additionally provide continuous high-precision longitudinal movement for FBCT acquisition and ensure highly accurate and reproducible patient positioning during transfers between RT and CT centers. Existing QA protocols based on AAPM TG-142 recommendations are inadequate for comprehensively validating these advanced functionalities. To address this problem, this study develops methodologies that integrate AAPM TG-142 and TG-66 recommendations with specific consideration of the CT linac’s unique system architecture and clinical workflow requirement and conducts a systematic mechanical performance evaluation of the novel treatment couch.

The couch top fully complies with all AAPM TG-66 specifications regarding levelness and perpendicularity of its longitudinal movement relative to the CT imaging plane. Furthermore, the system demonstrates complete adherence to TG-66 and TG-142 requirements for both directional/positional accuracy in translational movements and precision in rotational adjustments. This comprehensive compliance ensures reliable performance across all clinical functions, from diagnostic-quality CT acquisition to precise setup error correction during treatment delivery.

The measurements of rigidity demonstrated that the couch top exhibited varying degrees of sag and pitch angle under different load conditions and extension lengths. The uLinac VisionaryTx couch top demonstrated rigidity superior to that of the dedicated CT simulator but slightly inferior to the conventional linac couch top, yet it remained compliant with the IEC-60976 standard requirements (13). This results from its longer travel range and a deliberate design choice to reduce thickness for lower beam attenuation, which consequently compromises rigidity. In clinical practice, the couch sag and pitch angle variation resulting from this limited rigidity primarily affect treatment accuracy in two aspects: first, after completing the “Go Zero” correction to clear the residual pitch and roll angles from the previous patient’s 6D correction, if long-range couch top movement is still required to align the patient’s surface markers with the laser lights, additional couch top sag or pitch angle may be introduced during this long-range movement due to changes in the couch extension distance. If IGRT is not used for this fraction, even with perfect alignment at the isocenter, this pitch angle can cause significant positioning errors at both ends of an elongated target volume. To minimize additional couch top sag and pitch angle, the”Go Zero” operation should be performed after the Patient has been moved as close to the treatment Isocenter as practicable. Second, during continuous couch translation for CT acquisition, changes in extension length resulted in up to 1.3 mm of additional sag at the isocenter. Therefore, continuous monitoring of the couch top position is required, with corrections applied during image reconstruction.

In the FBCT IGRT workflow of the uLinac VisionaryTx system, patient transfer between the treatment (RT) and CT positions is accomplished solely through longitudinal movement of the couch base, eliminating the need for coordinated couch top motion. This effectively avoids introducing additional errors associated with couch top movement (such as couch top sag), while simultaneously imposing stringent requirements on the positional accuracy of the couch base. The couch base achieves positional accuracy and reproducibility better than 1 mm during longitudinal movement, ensuring precise patient positioning between RT and CT centers. Furthermore, the system exhibits excellent homing accuracy and repeatability in isocentric rotation and maintains exceptional coaxial alignment between the longitudinal movements of the couch base and couch top. These mechanical precision consistently maintains perpendicularity between the couch top’s longitudinal movement and the CT imaging plane during each FBCT IGRT procedure, thereby preventing CT image distortion. It should be emphasized that, while both AAPM TG-142 and MPPG 8.b (14) specify a tolerance of < 1°for couch absolute position readout, this may be insufficient for CT-Linac systems. Such tolerances may insufficiently maintain perpendicular alignment between couch top’s longitudinal movement and CT imaging plane, potentially resulting in geometric distortions in reconstructed CT images. It is therefore imperative to implement stricter tolerance for couch absolute position accuracy for CT-Linac systems.

Moreover, a significant couch top sag of up to 4 mm was observed during the transition from the RT center to CT center. This sag exhibits load-dependent characteristics, making it difficult to be corrected using a pre-defined model. Without proper compensation, such sag would introduce non-negligible errors into image registration results. The laser rangefinder based correction method proposed by UIH has been validated to effectively compensates for this couch sag error across various loading conditions, with residuals between calculated and measured displacements (ΔD1 and ΔD2) remaining below 0.6 mm. Although this study found that the laser rangefinder exhibits excellent short-term and long-term stability in both distance measurement and angular alignment, we recommend implementing regular QA protocol to monitor its measurement accuracy and angular stability, given their critical impact on correction performance.

End-to-end test serves as a critical verification method in radiotherapy QA protocols, to evaluate the comprehensive accuracy and reliability of complete clinical workflows. After systematically evaluating the mechanical performance of individual couch components, this study conducted comprehensive end-to-end testing of IGRT workflows under clinically representative conditions, including varying loading (30 kg - 135 kg), couch top extension (40 cm - 133 cm) and reference image sources. The results demonstrate clinically satisfactory IGRT accuracy across all tested conditions with translational precision superior to 0.5 mm and rotational accuracy better than 0.5°. The excellent end-to-end test results are ensured by two factors: first, the accuracy of imaging and registration (see **Table 3**); second, the couch relative movement constancy, which fully meets the requirements of AAPM MPPG 8.b (see section 3.1). This robust performance across diverse clinical scenarios provides compelling evidence of the treatment couch system’s exceptional mechanical capabilities and their successful integration into clinical workflows.

Driven by the objectives of enhancing therapeutic efficiency and integrating advanced imaging modalities, recent years have witnessed the emergence of novel accelerator designs (15–17) featuring co-axial non-coplanar configuration of positioning, treatment, and imaging centers. The comprehensive QA methodology developed in this study provides a valuable framework for mechanical performance validation of these advanced radiotherapy systems.

## 5 Conclusion

The novel treatment couch system of the uLinac VisionaryTx CT linac demonstrates exceptional mechanical performance that fully satisfy the precision requirements for CT acquisition, image guidance, and setup error correction, thereby ensuring high precision treatment delivery. For acceptance testing and ongoing QA, it is essential to incorporate additional specific QA measures beyond conventional protocols, based on AAPM TG-142 and TG-66 reports while accounting for its unique characteristics.

## Data Availability

The raw data supporting the conclusions of this article will be made available by the authors, without undue reservation.
